# Ezrin is required for adhesion and migration in invasive breast cancer

**DOI:** 10.1186/1753-6561-7-S2-P4

**Published:** 2013-04-04

**Authors:** Alvin Szeto, Victoria Hoskin, Bruce E Elliott

**Affiliations:** 1 Division of Cancer Biology and Genetics, Queen’s Cancer Research Institute, Kingston ON, Canada; 2 Department of Pathology and Molecular Medicine, Queen’s University, Kingston, ON, Canada

## Background

Deregulation of focal adhesion (FA) dynamics has been found to contribute to tumour progression by promoting cancer cell migration and invasion. Both focal adhesion kinase (FAK) and Src are involved in FA formation, and both interact with the scaffolding protein ezrin, which our group has shown to be required for breast cancer metastasis. Our aim was to assess the role of the membrane-cytoskeletal linker protein ezrin in Src-induced adhesion and migration in the human invasive breast cancer cell line MDA-MB-231.

## Materials and methods

shRNA-mediated knockdown (KD) of ezrin function was conducted in MDA-MB-231 cells, as well as in cells expressing constitutively active Src (MDA-Src). Adhesion and migration were assessed using collagen-I adhesion assays and wound healing assays, respectively. Expression and localization of FA proteins was assessed via immunoblotting and immunofluorescence (IF).

## Results

Ezrin KD impaired migration of both MDA-MB-231 and MDA-Src cells. Interestingly, Ezrin KD resulted in increased cell adhesion to collagen-I, an extracellular matrix protein found in stromal tissue. IF imaging of the FA-associated proteins β1 integrin, FAK, paxillin, and vinculin revealed an increase in localization of these proteins to FA sites at the cell periphery in ezrin KD cells compared to control cells. Furthermore, immunoblotting analysis showed increases in protein expression of β1 integrin, FAK and paxillin but not vinculin, suggesting that FA formation may be increased when ezrin expression is abolished.

## Conclusions

Ezrin is required for adhesion and migration of MDA-MB-231 cells, and may play an important regulatory role in FA dynamics.

## Financial support

Canadian Institutes of Health Research (BEE), Ontario Graduate Scholarship (AS), Terry Fox Foundation Training Studentship in Transdisciplinary Cancer Research (AS) and Canadian Breast Cancer Foundation Fellowship (VH).

